# Elevated Plasma Level of Soluble Form of RAGE in Ischemic Stroke Patients with Dementia

**DOI:** 10.1007/s12017-017-8471-9

**Published:** 2017-11-02

**Authors:** Sung-Chun Tang, Kai-Chien Yang, Chaur-Jong Hu, Hung-Yi Chiou, Chau Chung Wu, Jiann-Shing Jeng

**Affiliations:** 1 Department of Neurology, National Taiwan University Hospital, Taipei, Taiwan; 2 Departments of Internal Medicine, National Taiwan University Hospital, Taipei, Taiwan; 3 Department of Neurology, Taipei Medical University Hospital and Shuang Ho Hospital, Taipei, Taiwan; 4 School of Public Health, Taipei Medical University, Taipei, Taiwan

**Keywords:** Ischemic stroke, Vascular dementia, sRAGE, esRAGE

## Abstract

The receptor for advanced glycation end products (RAGE) and its downstream pathways are involved in various inflammatory and immune responses. Importantly, there is soluble RAGE (sRAGE) that forms either by alternative splicing of RAGE messenger ribonucleic acid as the endogenous soluble form of RAGE (esRAGE) or by proteolytic cleavage of full-length RAGE protein. This study aimed to investigate the associations of the plasma levels of sRAGE and esRAGE in ischemic stroke (IS) patients with and without dementia. This cross-sectional study recruited patients with IS at a university medical center. Vascular dementia was defined as the scale of Clinical Dementia Ranking (CDR) ≥ 1. Standard enzyme-linked immunosorbent assay was used to measure the plasma concentration of sRAGE and esRAGE. From November 2014 to October 2015, a total of 172 IS patients (mean age: 72.1 ± 7.5 years, 64.5% male) were recruited, including 73 with CDR = 0, 63 with CDR = 0.5, and 36 with CDR ≥ 1. In univariate analysis, IS patients with dementia were older and had more diabetes mellitus, less atrial fibrillation, and higher post-stroke modified Rankin Scale scores than those without dementia. Plasma levels of sRAGE and esRAGE were significantly higher in IS patients with than those without dementia (1.44 ± 1.29 vs. 1.03 ± 0.48 and 0.39 ± 0.40 vs. 0.24 ± 0.13 ng/mL, both *p* < 0.01). Importantly, both parameters remained independent after adjustment for clinical variables (OR 2.683, *p* = 0.013 and OR 39.192, *p* = 0.006, respectively). In summary, plasma sRAGE and esRAGE were elevated in those with dementia compared with those without dementia among IS patients.

## Introduction

Stroke is the third leading cause of death worldwide and the most frequent cause of permanent disabilities (Bhatnagar et al. 2010). Although stroke mortality has declined in recent years, the increased survival rates may translate into an increased number of cognitively impaired stroke patients (Pendlebury and Rothwell 2009). Vascular dementia (VD) is one of the most common causes of dementia. Around 10% of stroke survivors may develop dementia within 3 months after stroke, and an additional 20% develop dementia during the subsequent 3 years (O’Brien and Thomas 2015). However, whether there are certain post-stroke pathophysiological mechanisms specifically involving the development of VD remains controversial (Thiel et al. 2014; Pugazhenthi et al. 2017).

The receptor for advanced glycation end products (RAGE) is located on the plasma membrane of many types of cells including neurons (Alexiou et al. 2010). A variety of ligands, including the advanced glycation end products (AGE), high-mobility group box 1 (HMGB1), binds to RAGE. Importantly, there is soluble RAGE (sRAGE) that forms either by alternative splicing of RAGE messenger ribonucleic acid as the endogenous soluble form of RAGE (esRAGE) or by proteolytic cleavage of full-length RAGE protein (Kim et al. 2005). Biological studies have demonstrated that sRAGE may have diverse roles reflecting the activation of cellular RAGE signaling and also functioning as a decoy to compete with membrane RAGE for ligand binding (Lotze and Tracey 2005).

Recently, RAGE has been shown to be involved in the neurodegenerative pathways in Alzheimer’s disease (AD) (Pugazhenthi et al. 2017; Gasiorowski et al. 2017). A few studies have found decreased levels of plasma sRAGE in patients with AD compared to those with VD or non-demented controls (Qian et al. 2012; Xu et al. 2017; Liang et al. 2013; Emanuele et al. 2005). However, none of these studies directly compared the difference between stroke patients with or without dementia. In our study, we measured plasma levels of sRAGE and esRAGE in patients with ischemic stroke (IS) and correlated the results with neurocognitive status.

## Materials and Methods

### Study Design and Population

This prospective case series cross-sectional study was conducted at the National Taiwan University Hospital (NTUH), with the approval of the Research Ethics Committee of NTUH. Written informed consent was obtained from the patients or from their next of kin without a significant cognitive impairment.

Patients with a history of IS who had been receiving regular follow-up at the Neurology Outpatient Clinic from November 2014 to October 2015 were recruited. The diagnosis of IS was confirmed and characterized by head MRI or CT examinations (performed at least 24 h after stroke onset to clearly demonstrate the infarct region). Patients with a known active infection, cancer, renal disease (creatinine > 2.0 mg/dL), autoimmune disorder, current steroid treatment, or poor diabetes control (hemoglobin A1c > 8.0%) were excluded. A detailed history of stroke, vascular risk factors, and comorbidity was obtained from each patient. Body mass index (BMI) was calculated as weight divided by the square of height.

### Dementia Diagnosis and Severity Rating

Clinical diagnoses of VD were made by neurologists in accordance with internationally accepted criteria for dementia (National Institute of Neurological Disorders and Stroke and Association Internationale pour la Recherché et l’Enseignement en Neurosciences [NINDS-AIREN]) (Tierney et al. 1989; Roman et al. 1993). Dementia severity was measured by the Clinical Dementia Rating (CDR) scale. The Mini–Mental State Examination (MMSE) and Montreal Cognitive Assessment (MoCA) testing was performed as part of the clinical evaluation. Post-stroke outcome was defined by a modified Rankin Scale (mRS) score.

### Blood Sampling

#### Human Plasma Collection and Measurements

Blood samples were drawn at the time of recruitment. A 10-mL sample of blood was drawn, then centrifuged at 300 g for 15 min, and subsequently aliquoted into 1.5-mL tubes and stored at − 80 °C until ready for use. The plasma levels of total sRAGE and esRAGE were determined using a commercially available enzyme-linked immunoassay kit (RAGE: R&D Systems, Minneapolis, MN, esRAGE Human ELISA Kit, B-Bridge International, Cupertino, CA) according to the manufacturer’s protocol. Measurements were taken in duplicate, and the results were averaged. Samples with obvious hemolysis, which was visually detected by a pink to red tinge inside, were not used for measurements.

### Statistical Analyses

Statistical analyses were performed using SPSS version 17.0 software (Chicago, IL). The clinical characteristics of VD and non-VD patients were compared using the Fisher’s exact test, Chi-squared test, Student’s *t* test, and Mann–Whitney *U* test with relevant variables as indicated. Multivariable logistic regression analysis models were used with VD as the dependent variable. The independent variables in the analyses were age, gender, diabetes mellitus, hypertension, atrial fibrillation, and post-stroke mRS. Figures were made using GraphPad Prism 4.0 software, and data were expressed as mean ± SEM. Comparisons between subgroups were made with Student’s t test, logistic regression, and ANOVA followed by Newman–Keuls post hoc analysis.

## Results

### Study Subject Demographics

During the study period, consecutive patients who were regularly followed up at the Neurology Outpatient Clinic of NTUH and fulfilled the study criteria were recruited. A total of 172 IS patients (64.5% male) were included in our study. The average time interval between stroke onset and recruitment into the study was 85.0 ± 54.0 months. The clinical information for normal and mild cognitive impairment (CDR ≦ 0.5, *n* = 136) and VD patients (CDR ≧ 1, *n* = 36) is listed in Table 1. Compared to IS patients without dementia, those with dementia were older, had more diabetes mellitus and less atrial fibrillation, lower mean scores on the MMSE and MoCA, a lower rate of functional independence, and a longer time interval between stroke onset to study recruitment (all *p* < 0.05).

**Table 1:** Clinical parameters and plasma levels of sRAGE and esRAGE in stroke patients with and without vascular dementia

	CDR ≦0.5 (*n* = 136)	CDR ≧1 (*n* = 36)	*p*
Age (years)	71.2 ± 6.9	75.4 ± 8.8	0.002
Male, n	90 (66.2)	21 (58.3)	0.435
Interval between stroke and recruitment (months)	80.2 ± 52.5	102.1 ± 56.3	0.031
Diabetes mellitus	61 (46.2)	11 (30.5)	0.061
Hypertension	111 (81.6)	31 (86.1)	0.776
Hyperlipidemia	75 (55.1)	20 (55.5)	0.704
Smoking	46 (33.8)	17 (47.2)	0.252
CAD	17 (12.5)	5 (13.9)	0.245
AF	12 (8.8)	0 (0)	0.070
Carotid stenosis ≧ 50%	20 (14.7)	5 (13.9)	1.000
MRS	1.29 ± 0.92	2.72 ± 1.09	< 0.001
MMSE	25.7 ± 3.5	15.2 ± 5.6	< 0.001
MoCA	19.8 ± 5.2	10.5 ± 6.8	< 0.001
sRAGE (ng/ml)	1.03 ± 0.48	1.44 ± 1.29	0.003
esRAGE (ng/ml)	0.24 ± 0.13	0.39 ± 0.40	< 0.001

*CDR* Clinical Dementia Ranking, *CAD* coronary artery disease, *AF* atrial fibrillation, *MRS* modified Rankin Scale, *MMSE* Mini–Mental State Examination, *MoCA* Montreal Cognitive Assessment, *sRAGE*, soluble form of receptor for advanced glycation end products, *esRAGE* endogenous soluble form of receptor for advanced glycation end products

### RAGE Signaling-Related Plasma Biomarkers in Study Subjects

**Fig. 1:**
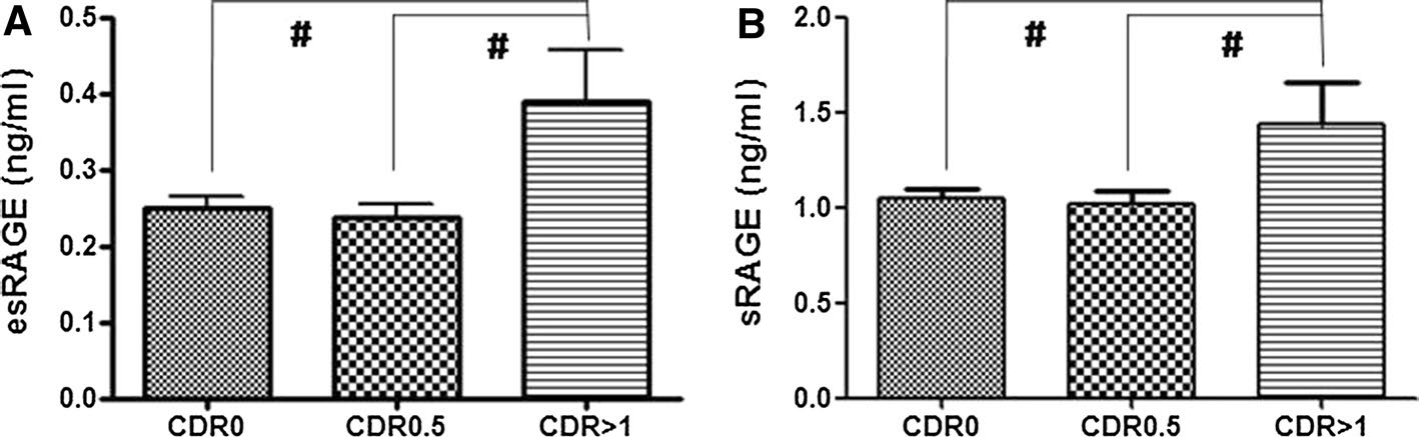
Plasma levels of esRAGE (**a**) and sRAGE (**b**) in ischemic stroke patients with normal cognitive function (CDR = 0), mild cognitive impairment (CDR = 0.5), and dementia (CDR = 1). It showed that levels of esRAGE and sRAGE were significantly higher in ischemic stroke patients with dementia compared to those without dementia (both *p* < 0.001)

Figure 1 shows plasma levels of sRAGE and esRAGE in IS patients without normal cognitive function, mild cognitive impairment, and dementia. In univariate analysis, plasma levels of sRAGE and esRAGE were higher in IS patients with dementia compared to those without dementia (both *p* < 0.001). After adjustment for clinical parameters including age, gender, diabetes mellitus, hypertension, atrial fibrillation, interval between stroke onset to study recruitment, and post-stroke mRS, plasma levels of sRAGE and esRAGE remained significantly higher in stroke patients with dementia compared with those without dementia [odds ratio 2.65 (1.21–5.79) and 35.90 (2.65–485.53), *p* = 0.014 and 0.007, respectively].

## Discussion

In our study, we aimed to clarify the difference between IS patients with and without dementia. We recruited patients with a history of IS, collected blood samples to measure the levels of sRAGE and esRAGE, and evaluated cognitive functional status at the same time. Our results suggested that IS patients with dementia had significantly higher levels of sRAGE and esRAGE compared to those without dementia. These findings may indicate the direct involvement of intracellular RAGE signaling in the development of VD.

A review of the previous literature revealed that levels of serum or plasma sRAGE in patients with VD had been investigated in only a few studies. In 2005, Emanuele et al. reported that levels of sRAGE in patients with AD (*n* = 152) were significantly lower than those with VD (*n* = 91) and controls (*n* = 161) (Emanuele et al. 2005). In 2012, Qian et al. recruited patients with acute stroke (*n* = 152) (Qian et al. 2012). Blood sample was collected at the time of admission, and cognitive function was evaluated at 2 weeks after stroke. The results showed that serum levels of sRAGE at the time of stroke onset were lower in patients with post-stroke cognitive impairment than in those without post-stroke cognitive impairment. In 2013, Ling et al. measured the plasma levels of sRAGE in patients with AD (*n* = 126), VD (*n* = 96), and cognitively normal controls (*n* = 98) (Liang et al. 2013). The results showed that levels of sRAGE were significantly lower in the group of AD compared with the group of VD patients and the controls. There was no difference in sRAGE levels between patients with VD and the controls. In 2016, Xu et al. measured sRAGE concentration in 36 patients with AD, 12 with VD, and 35 with cognitively normal controls, and the data showed that levels of sRAGE were not statistically different among these three groups (Xu et al. 2017). Overall, serum/plasma levels of sRAGE might be lower in patients with AD compared to other types of dementia and normally cognitive controls. Whether levels of sRAGE in patients with VD are lower than in non-AD subjects is uncertain. In addition, none of the studies directly compared the levels of sRAGE between stroke patients with and without dementia. Therefore, we recruited only stroke patients with and without dementia, and thus, the data of clinical and biological parameters should be able to reflect the factor of dementia after statistical analysis. It is important to mention that this had not been addressed previously in other studies. In our study, although there were some differences in clinical parameters between the IS patients with and without dementia, levels of sRAGE and esRAGE remained statistically significant after adjustment for clinical parameters.

In our previous studies, we measured the levels of sRAGE in patients with acute stroke within 48, 48–72 h, and 5–7 days after stroke onset (Tang et al. 2013, 2016). The data suggested that the level of sRAGE has a dynamic pattern after the onset of stroke from initially high to lower through the acute to subacute phases of stroke. In addition, higher levels of sRAGE in stroke within 48 h after onset are significantly associated with unfavorable functional outcome. Therefore, we proposed that circulating sRAGE in acute stroke may mainly reflect the activation of cellular RAGE rather than function as a decoy receptor. Similarly, our results of higher levels of plasma sRAGE in IS patients with dementia than in those without dementia may indicate the activation of intracellular RAGE signaling-mediated inflammatory response in the development of dementia after stroke.

Importantly, not only sRAGE levels, but also levels of esRAGE were elevated in our IS patients with dementia. The significant difference between IS patients with and without dementia was even greater than for sRAGE after multivariant analysis. Since esRAGE is produced via the alternative splicing of RAGE messenger ribonucleic acid, this finding not only supports the finding that sRAGE increased in IS patients with dementia, but also further strengthens the importance of endogenous RAGE signaling activation in the pathophysiological mechanism of VD.

Our study has several limitations. First, this is a single hospital study, and the possibility of case selection bias cannot be ignored. Second, this is a case series cross-sectional study, so only association but not causal relation between sRAGE and VD can be established from our study result. Besides, it is also possible that our recruited cases may have mixed dementia or already have cognitive impairment before the onset of stroke. Further study with a prospective cohort study design may help to clarify this issue. Third, our study is a purely clinical observation and can only present the data based on the measured values. Whether the levels of sRAGE and esRAGE are reflecting the activity of intracellular RAGE signaling, as we proposed, requires further investigation. Theoretically, it would have been informative to investigate the expression of RAGE protein in the brain between IS patients with or without dementia and correlate the results with plasma levels of sRAGE. Nevertheless, our study did demonstrate a novel phenomenon that plasma levels of sRAGE and esRAGE increased in stroke patients with VD compared to those without VD.

In conclusion, among IS patients, plasma levels of sRAGE and esRAGE were elevated in those with dementia.

## Abbreviations

VD: Vascular dementia (VD)
RAGE: Receptor for advanced glycation end products
sRAGE: Soluble form of RAGE
esRAGE: Endogenous soluble form of RAGE
AD: Alzheimer’s disease
IS: Ischemic stroke
CDR: Clinical Dementia Ranking
ELISA: Enzyme-linked immunoassay
CAD: Coronary artery disease
AF: Atrial fibrillation
MRS: Modified Rankin Scale
MMSE: Mini–Mental State Examination
MoCA: Montreal Cognitive Assessment
